# *In-situ* syntheses of graft copolymers by metal-free strategies: combination of photoATRP and ROP

**DOI:** 10.1080/15685551.2020.1808414

**Published:** 2020-08-18

**Authors:** Gorkem Yilmaz

**Affiliations:** Department of Chemistry, Istanbul Technical University, Maslak, Turkey

**Keywords:** Photochemistry, controlled/living radical polymerization, atom transfer radical polymerization, ring-opening polymerization, graft copolymers

## Abstract

A completely metal-free and environmentally friendly strategy is demonstrated for the preparation of graft copolymers by combining photoinduced Atom Transfer Radical Polymerization (ATRP) and Ring Opening Polymerization (ROP). Polymerizations are simultaneously realized in a one-pot manner. For this purpose, bare vinyl monomers, vinyl monomers with hydroxyl functional groups, and lactone monomers were simultaneously polymerized under visible light using specific catalysts. While vinyl monomers construct the main chain, the lactone monomers were polymerized from the hydroxyl functions present at the side chain. Spectral and chromatographic analyses prove that the utilized strategy is successful in the preparation of graft copolymers controlled molecular weights and narrow distributions.

## Graphical abstract

**Figure:**
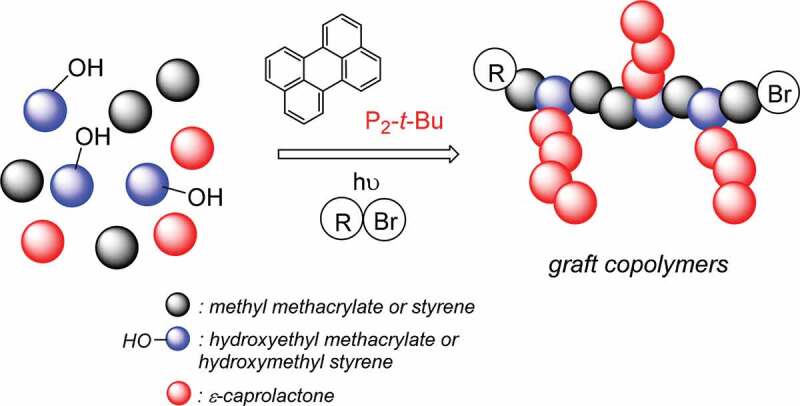

## 1. Introduction

Discovery of controlled/living polymerization (CLP) methods have brought a fresh air to the synthetic polymer chemistry due to their unique advantages in comparison to the conventional techniques [1–7]. They provide synthesis of polymers with controlled molecular weight characteristics and functional groups. In addition, due to the lack of termination and transfer reactions, polymerizations can theoretically reach up to complete conversion that cannot be achieved with traditional paths. These considerable advantages pave way for researchers to utilize CLP for the syntheses of complex macromolecular structures such as block, graft and star copolymers and hyperbranched polymers [8,9].

Among the CLP techniques studied, atom transfer radical polymerization (ATRP) is the most intensively investigated tool for the polymerization of vinyl monomers [1,2]. Traditional ATRP generally requires low oxidation state copper complexes as catalysts that mediate the process. Attempts have been focused on decreasing the required concentration of these complexes, which include simultaneous chemical reduction10 and photochemical methodologies [10–17]. More recently, several photocatalysts have been developed that mediate ATRP even in the absence of inorganic complexes [18–25]. This so-called photoinduced metal-free ATRP has been employed for the surface modification [26,27] and syntheses of various polymeric structures including block copolymers [28], star copolymers [29] and hyperbranched polymers.

Graft copolymers are segmented copolymers in which homopolymeric branches of one polymer type are distributed randomly on a linear main-chain polymer of another type [6,30–32]. They display physicochemical properties different than the constructing segments especially due to the incompatibility of the segments when they are chemically unlike. Therefore, graft copolymers find practical applications such as impact-resistant materials, thermoplastic elastomers, compatibilizers or emulsifiers for the preparation of stable blends or alloys, and preparation of biodegradable plastics or films.

**Scheme 1.**
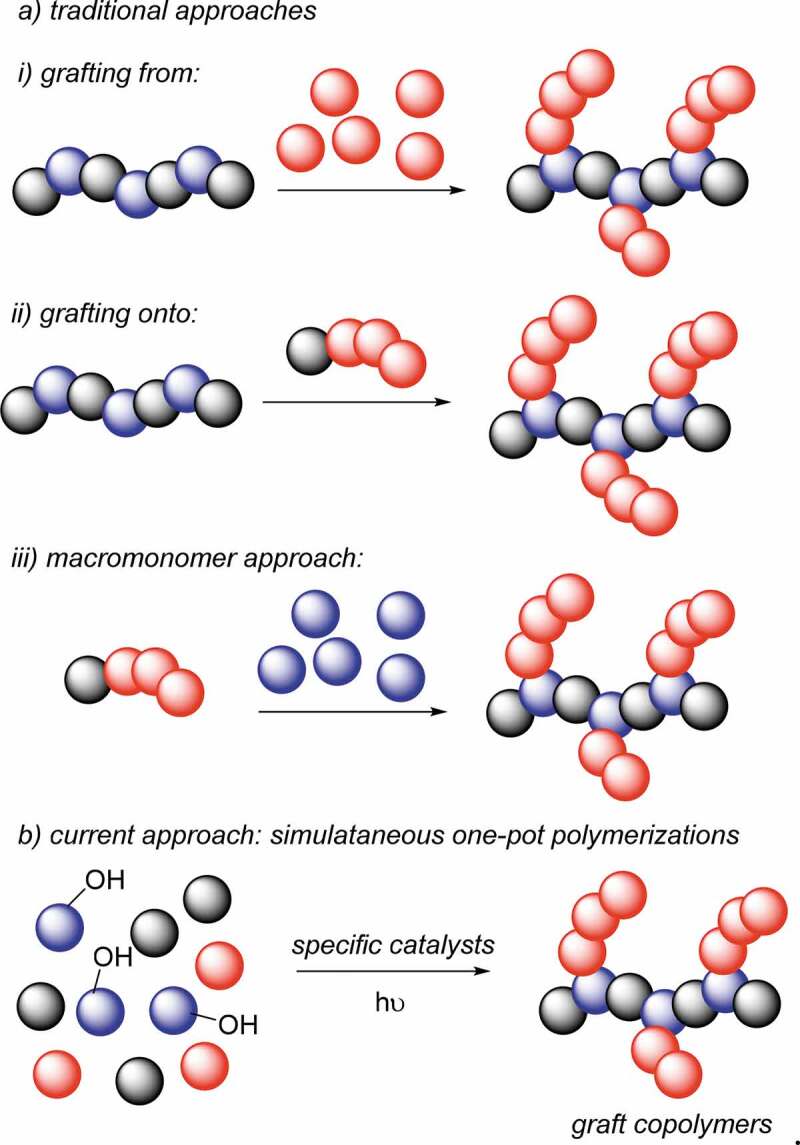
Traditional approaches (a) and current approach (b) for the synthesis of graft copolymers

Graft copolymers can be synthesized generally by three main approaches; (*i*) the grafting-from approach, which considers the formation of branch polymers from an active center generated on the main chain, (*ii*) the grafting-onto approach where the side chains are individually prepared and attached to a main chain and (*iii*) the macromonomer method, which is realized by copolymerizing a macromolecule having polymerizable functionalities at the chain end (Scheme 1a).

As a part of our continuing interest in the preparation of complex macromolecular architectures by combining metal-free strategies, herein we report a one-pot strategy for graft copolymer syntheses (Scheme 1b). Our strategy is based on the combination of photoinduced metal-free ATRP with ring opening polymerizations (ROP) in a simultaneous manner. The polymerizations are conducted in metal-free conditions at room temperature. In this sense, this strategy provides a completely environmentally friendly way to prepare graft copolymers in a very fast fashion. In addition, the preparation and purification steps are reduced to a single step, which provides less chemical consumption in comparison to traditional ways for graft copolymer syntheses.

## 2. Materials and methods

### 2.1. Materials

Methyl methacrylate (MMA, 99%; Aldrich), 2-hydroxyethyl methacrylate (HEMA, 99%; Aldrich) and styrene (St, 99%, Aldrich) were passed through a basic alumina column to remove the inhibitor. 4-hydroxymethyl styrene (HMS) was synthesized from 4-vinylbenzyl chloride (90%; Acros Organics) as reported and then vacuum distilled [33]. ε-Caprolactone (CL, 97%; Aldrich) was distilled under vacuum over CaH_2_. Ethyl α-bromophenylacetate (EBPA, 97%; Alfa Aesar), phosphazene base (P_2_*-t-*Bu, ~2.0 M in THF; Aldrich) and perylene (>98.0% (GC); TCI) was used as received. Toluene (99.5%; Aldrich) was distilled over sodium and benzophenone as indicator and stored in molecular sieves under nitrogen atmosphere. Methanol (Technical) and n-hexane (≥95.0% (GC); Merck) were used to precipitate polymers.

### 2.2. Characterization

^1^ H-NMR spectra were recorded at 500 MHz on an Agilent VNMRS 500 spectrometer at room temperature. Gel permeation chromatography (GPC) measurements were performed on a TOSOH EcoSEC GPC system equipped with an autosampler system, a temperature-controlled pump, a column oven, a refractive index (RI) detector, a purge and degasser unit and TSKgel superhZ2000, 4.6 mm ID x 15 cm x 2 cm column. Tetrahydrofuran was used as an eluent at ﬂow rate of 1.0 mL/min at 40°C. Refractive index detector was calibrated with polystyrene standards having narrow molecular weight distributions. Data were analyzed using Eco-SEC Analysis software. Fourier transform infrared (FTIR) spectra were recorded on Perkin−Elmer FTIR Spectrum One spectrometer with an ATR Accessory and cadmium telluride detector. UV-visible spectra were recorded with a Shimadzu UV-1601 spectrometer. DSC measurements were performed on Perkin-Elmer Diamond DSC with a heating rate of 10°C min^−1^ under nitrogen flow.

### 2.3. General route for concurrent photoinduced metal-free ATRP and ROP

HEMA (5 eq), MMA (200 eq), CL (95 eq), perylene (3 eq), EBPA (1 eq), P_2_*-t-*Bu (1 eq) and toluene were taken into a flame dried Schlenk tube. The tube was purged with nitrogen and sealed tightly. The reaction mixture was irradiated at 400–500 nm with a light intensity of 45 mW cm^−2^ for given time intervals. Resulted polymers were precipitated in cold methanol and dried under pressure.

### 2.4. Sequential polymerization of polymers; first photoinduced metal-free ATRP of MMA and HEMA, then sequential addition of CL by metal-free ROP

A flame dried Schlenk tube was filled with HEMA (5 eq), MMA (200 eq), perylene (2 eq), EBPA (1 eq) and toluene (V_MMA_/Vtoluene: 1/7). After the reaction mixture was degassed with pure nitrogen gas, the tube was covered tightly and irradiated for 4 h at λ = 400–500 nm. Reaction mixture was precipitated into excess hexane. Precipitated polymers were filtered and dried in vacuum.

In the second step, the precursor polymer (1 eq), CL (800 eq), P_2_*-t-*Bu (40 eq) and toluene (V_CL_/V_toluene_: 1/1.5) was mixed in a flame dried Schlenk tube, purged with nitrogen and polymerization was carried out for 1 h. Resulted polymers were precipitated in hexane, filtered and dried under reduced pressure. Additionally, they were dissolved in THF and precipitated in diethyl ether methanol mixture (30/70 by volume) again.

## 3. Results and discussion

We used either methyl methacrylate (MMA)/hydroxyethyl methacrylate (HEMA) or styrene (S)/hydroxymethyl styrene (HMS) for main-chain construction and ε-caprolactone for side-chain formation. Perylene and phosphazene bases were used as catalysts for photoinduced metal-free ATRP and ROP processes, respectively. UV analyses reveal that there is no interaction between these catalysts, which enable their simultaneous usage in the same reaction medium (Supporting Information, SF1).

**Scheme 2.**
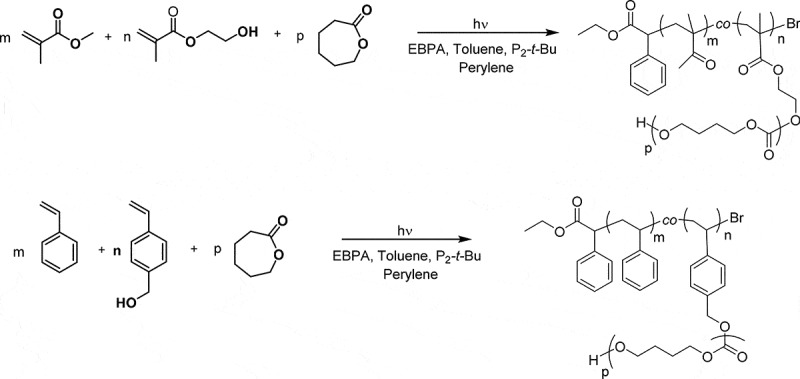
Syntheses of (PMMA-*co*-PHEMA)-*g*-PCL and (PS-*co*-PHMS)-*g*-PCL

The overall process for the synthesis of graft copolymers, namely (poly(methyl methacylate)-*co*-poly(hydroxyethyl methacrylate))-*g*-poly(ε-caprolactone) ((PMMA-*co*-PHEMA)-*g*-PCL) and (polysyrene-*co*-polyhydroxymethyl styrene)-*g*-poly(ε-caprolactone) ((PS-*co*-PHMS)-*g*-PCL) is given in Scheme 2.

The details of experimental conditions and the polymerization results are compiled in Table 1.

**Table 1.** Concurrent polymerization of CL, MMA and HEMA under different experimental conditions^a.^

Run	Monomers	Perylene (eq)	Irradiation Time (h)	*M*_n_^b^ (g·mol^−1^)	*M*_w_/*M*_n_^b^	Overall Conv.^d^ (%)
1	MMA/HEMA	3	1	3600	1.51	22.4
2	MMA/HEMA	3	2	4600	1.45	56.9
3	S/HMS	3	2	3900	1.22	30.0
4	S/HMS	4	2	4900	1.12	35.7

^a^HEMA or HMS/MMA or S/CL/EBPA: 5/95/200/1, V_MMA_ = 500 µL, V_tot_ = 3.9 mL (in toluene), λ ~ 400–500 nm. ^b^ Determined by gel permeation chromatography using polystyrene standards. ^c^ Calculated by ^1^ H NMR. ^d^ Determined gravimetrically.

Clearly, an increase in the irradiation time gives rise to an increase in the molecular weight of the graft copolymers, as well as the overall conversion as expected. When the concentration perylene is increased, the overall conversion was also found to increase due to the generation of higher concentration of active groups as expected.

**Figure 1.**
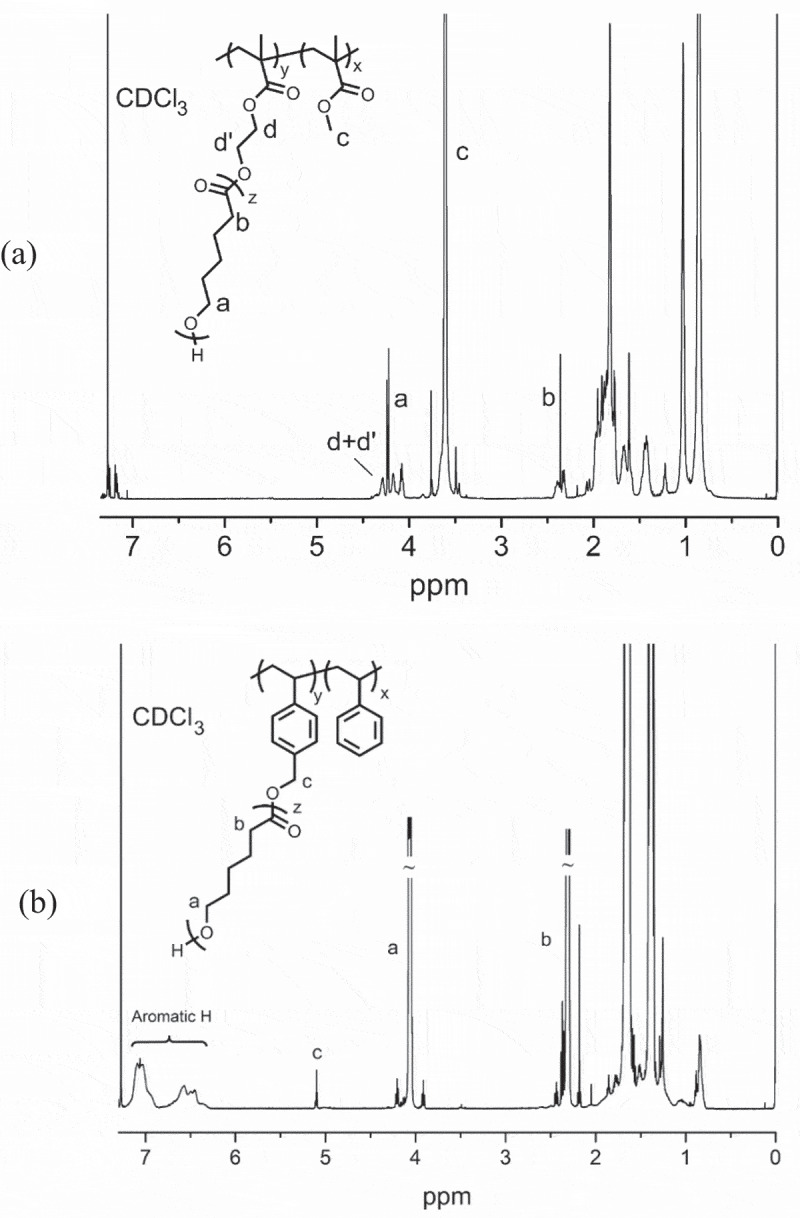
^1^ H NMR spectra of (PMMA-*co*-PHEMA)-*g*-PCL (a) and (PS-*co*-PHMS)-*g*-PCL (b)

The structures of the graft copolymers were investigated by ^1^ H NMR spectroscopy. Figure 1 shows the NMR spectra of the graft copolymers obtained. In Figure 1a, the peak around 3.6 ppm corresponds to the main-chain PMMA protons, while the peaks at 2.2 and 4.2 ppm belong to the protons of PCL segments at the side chain. The signals around 4.3 ppm can be attributed to the ester protons adjacent to the PCL segments. The NMR spectrum of (PS-*co*-PHMS)-*g*-PCL clearly shows the aromatic signals of the styrenic backbone around 6.5–7.2 ppm along with the main protons of the side chain PCL segments appeared at 2.2 and 4.2 ppm. The benzylic protons adjacent to the PCL segments were also observable at 5.1 ppm as below (Figure 1b).

**Figure 2.**
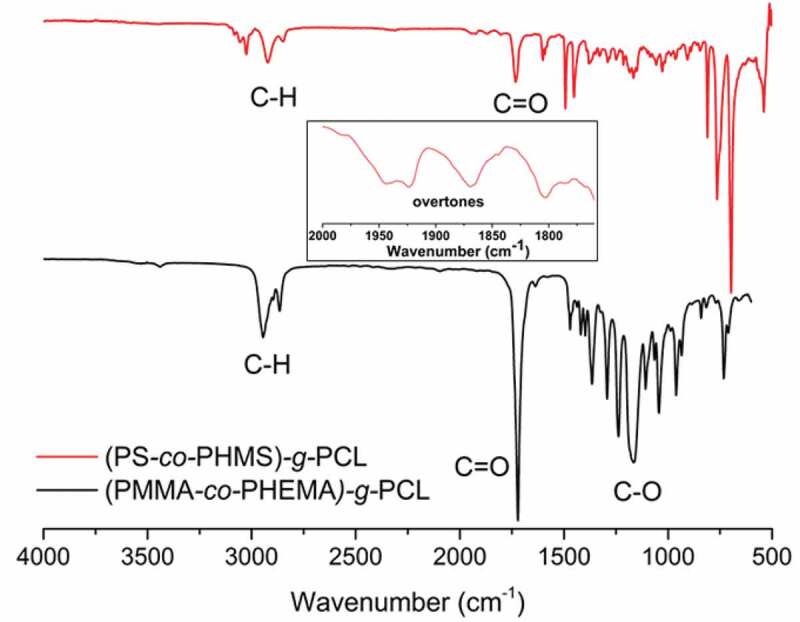
FT-IR spectra of (PMMA-co-PHEMA)-g-PCL (black line) and (PS-co-PHMS)-g-PCL (red line)

The structures of the graft copolymers were further investigated by FT-IR analysis. Figure 2 demonstrates the FT-IR spectra of (PMMA-*co*-PHEMA)-*g*-PCL (black line) and (PS-*co*-PHMS)-*g*-PCL (red line). The spectrum of (PMMA-*co*-PHEMA)-*g*-PCL displays a strong C = O stretching around 1735 cm^−1^, stemming from the ester groups of both PMMA and PCL segments. In addition, the strong C-O transitions around 1100 cm^−1^ further confirms the structure of the polymer. The FT-IR spectrum of (PS-*co*-PHMS)-*g*-PCL shows a weaker C = O stretching as less ester functions are present in its structure. The overtones around 1800–1950 cm^−1^ demonstrate the presence of aromatic groups in the polymer obtained.

**Figure 3.**
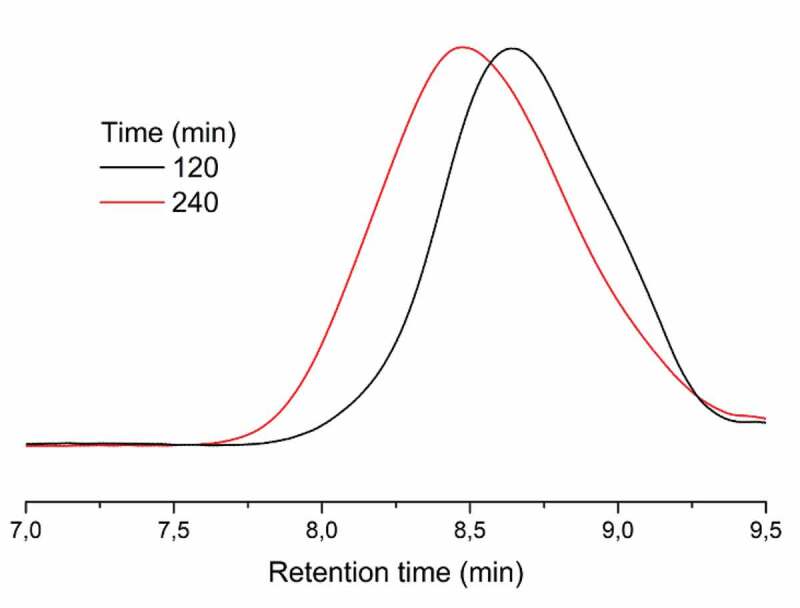
GPC traces of (PMMA-co-PHEMA)-g-PCL obtained in 2 and 4 h of irradiation

The dependence of irradiation time on the molecular weights of the polymers was investigated by GPC analysis (Figure 3). Clearly, when the irradiation time increases, the molecular weight of the graft copolymer is increased as expected. In addition, the unimodal chromatograms observed in both cases prove the success of the controlled polymerizations performed concurrently.

**Figure 4.**
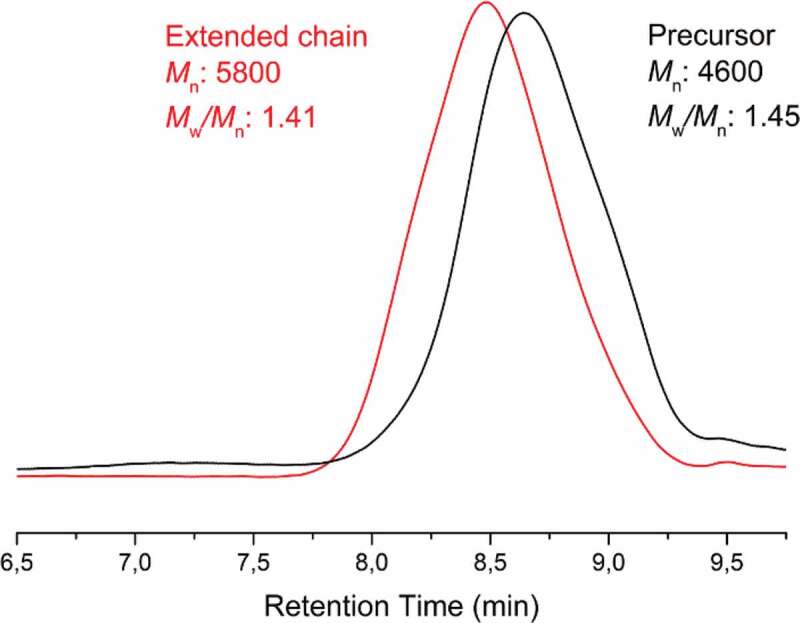
Comparison of GPC traces of precursor (PMMA-co-PHEMA)-g-PCL with chain-extended polymer by MMA

The chain-end fidelity of the polymers was tested by sequential polymerization. The obtained graft copolymer, namely (PMMA-*co*-PHEMA)-*g*-PCL, was used as a precursor for the polymerization of MMA by photoinduced metal-free ATRP. The bromide functional groups at the chain end act as initiating species for the growth of acrylic main-chain segment. GPC results show that there is a clear shift to the higher molecular weight region after the process (Figure 4).

**Figure 5.**
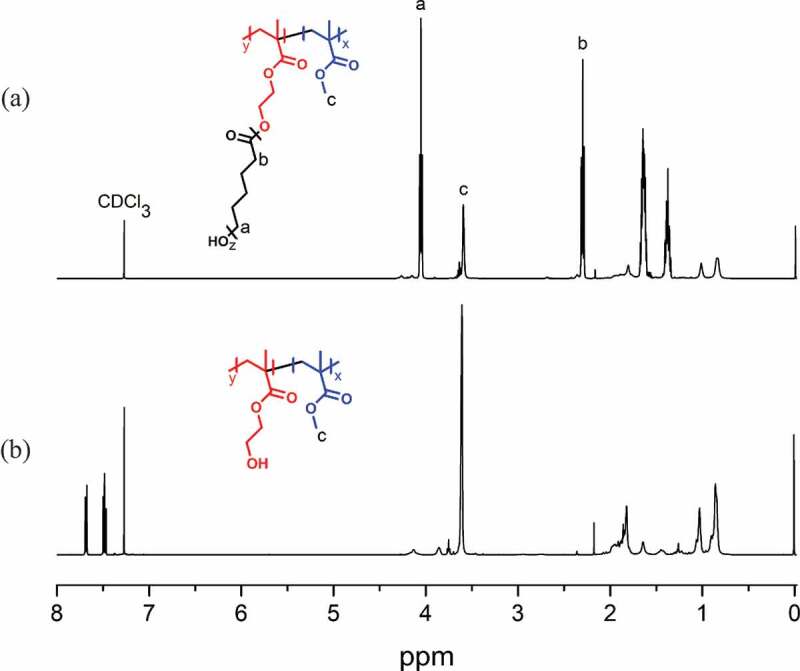
^1^ H-NMR spectra of PMMA-*co*-PHEMA and (PMMA-*co*-PHEMA)-*g*-PCL

To demonstrate the possibility of preparing these polymers in a sequential manner, first MMA and HEMA were copolymerized by photoinduced metal-free ATRP using perylene as photocatalyst. Then, thus obtained polymer, PMMA-*co*-PHEMA, was used as a precursor for the polymerization of CL to yield (PMMA-*co*-PHEMA)-*g*-PCL. Figure 5 shows the comparison of the NMR spectra of both polymers. The emergence of the new peaks at 2.2 and 4.2 ppm shows the success of the formation of PCL segments as expected (Figure 5a). The molecular weight analyses of each polymer can be found in the supporting information (SF2).

## 5. Conclusions

n conclusion, graft copolymers can be synthesized by the simultaneous combination of photoinduced metal-free ATRP and ROP. In comparison to traditional approaches applied, this strategy provides the preparation and purification of graft copolymers in a single step, which is of high importance by means of financial and ecological anticipations. This completely metal-free approach is shown to be applicable to the synthesis of graft copolymers with various monomer compositions. In addition, as it serves the incorporation of PCL segments in the macromolecular structure, this methodology is a useful tool for the synthesis of polymers employed for bio-applications. Further studies in this line are currently in progress.
